# Machine Learning Combined with Radiomics Facilitating the Personal Treatment of Malignant Liver Tumors

**DOI:** 10.3390/biomedicines12010058

**Published:** 2023-12-26

**Authors:** Liuji Sheng, Chongtu Yang, Yidi Chen, Bin Song

**Affiliations:** 1 Department of Radiology, West China Hospital, Sichuan University, Chengdu 610041, China; shenglj_1999@163.com (L.S.); henrys1011@163.com (C.Y.); 2 Functional and Molecular Imaging Key Laboratory of Sichuan Province, West China Hospital, Sichuan University, Chengdu 610041, China; 3 Department of Radiology, Sanya People’s Hospital, Sanya 572000, China

**Keywords:** machine learning, deep learning, radiomics, malignant liver tumors, personalized therapy

## Abstract

In the realm of managing malignant liver tumors, the convergence of radiomics and machine learning has redefined the landscape of medical practice. The field of radiomics employs advanced algorithms to extract thousands of quantitative features (including intensity, texture, and structure) from medical images. Machine learning, including its subset deep learning, aids in the comprehensive analysis and integration of these features from diverse image sources. This potent synergy enables the prediction of responses of malignant liver tumors to various treatments and outcomes. In this comprehensive review, we examine the evolution of the field of radiomics and its procedural framework. Furthermore, the applications of radiomics combined with machine learning in the context of personalized treatment for malignant liver tumors are outlined in aspects of surgical therapy and non-surgical treatments such as ablation, transarterial chemoembolization, radiotherapy, and systemic therapies. Finally, we discuss the current challenges in the amalgamation of radiomics and machine learning in the study of malignant liver tumors and explore future opportunities.

## 1. Introduction

In the arena of diagnosing, treating, and prognosticating malignant liver tumors, a convergence of cutting-edge medical imaging, data science, and machine learning (including artificial intelligence) has illuminated a promising path forward. This transformative innovation finds its embodiment in radiomics, an emerging field that combines the rich biological information contained in medical images with the powerful computational abilities of machine learning, shedding light on the intricate landscape of liver oncology [1,2].

Malignant liver tumors, encompassing hepatocellular carcinoma (HCC), intrahepatic cholangiocarcinoma (ICC), and liver metastases, present profound clinical challenges. While conventional imaging methods remain indispensable, they often struggle with the elusive subtleties of tumor heterogeneity, rendering the task of tailoring precise and personalized treatment strategies formidable. The field of radiomics rises to meet this challenge by extracting a wealth of quantitative features from medical images [2].

The study of radiomics stands as the forefront of the digital transformation of medicine, employing data-driven methodologies to unveil layers of information hidden within medical images, beyond human perception [2]. Advanced algorithms dissect images into thousands of attributes, including intensity, texture, and structural patterns [3]. Intensity features encapsulate gray-level histogram information, elucidating the global distribution of gray levels in images. Texture features delineate the relationships between adjacent voxels, while high-order features are derived through wavelet and Laplacian Gaussian filtering. Machine learning, a facet of artificial intelligence, facilitates data-driven learning for decision-making. Deep learning, a subset of machine learning, employs intricate artificial neural networks to discern complex patterns from extensive data [4]. These technologies empower the selection, analysis, and integration of radiomic features from diverse image types, enabling predictive models for a range of clinical applications. Radiomics has demonstrated its utility in lesion characterization, preoperative diagnosis, treatment efficacy evaluation, and prognosis prediction across different tumor models [5,6,7,8]. In the realm of malignant liver tumor treatment, numerous radiomics studies have harnessed multiparametric and multimodality imaging, achieving promising results [9,10,11,12,13,14,15,16,17,18,19,20,21,22,23,24,25,26,27,28,29,30,31,32,33,34,35,36,37,38,39,40,41,42,43,44,45,46,47,48,49,50,51,52].

In an era defined by the convergence of medicine and technology, radiomics, fortified by machine learning, holds the promise of redefining personalized treatment. This transformative synergy brings us closer to the realization of precision medicine, where each patient’s unique attributes and needs occupy the core of therapeutic strategies. This review details the development history, technical architecture, application status and future prospects of radiomics in promoting personalized treatment of patients with liver malignant tumor.

## 2. Evolution of Radiomics and Its Procedural Framework

**Figure 1:**
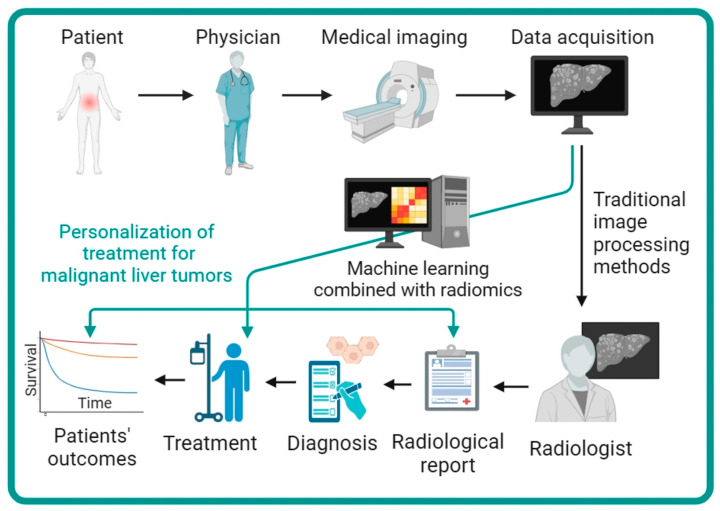
The workflow of machine learning combined with radiomics facilitating the personal treatment of malignant liver tumors. Compared to traditional imaging evaluation methods, radiomics analysis can provide a wealth of multidimensional information, thereby improving the diagnosis, treatment planning, and efficacy prediction for patients with malignant liver tumors.

Radiomics is a data-driven methodology for extracting a wide range of quantitative features from medical images, enabling an in-depth characterization of tumor patterns and traits that often elude human perception [2]. Its applications extend to enhancing the diagnosis, prognosis, and prediction of therapeutic responses across various medical conditions, with particular relevance in the field of oncology. Here, we searched PubMed (as of 12 September 2023) for radiomics studies pertaining to malignant liver tumors, using terms “hepatocellular carcinoma”, “HCC”, “cholangiocarcinoma”, “ICC”, “cholangiocellular carcinoma”, “liver”, “metasta*”, “liver cancer”, “machine learning”, “radiomics”. Our search identified 44 clinical target-oriented published works following manual screening [9,10,11,12,13,14,15,16,17,18,19,20,21,22,23,24,25,26,27,28,29,30,31,32,33,34,35,36,37,38,39,40,41,42,43,44,45,46,47,48,49,50,51,52]. The majority of these studies (31 out of 44) were performed in a single center within a retrospective cohort framework. Among the identified studies, the most commonly used imaging modality was computed tomography (CT) in 32 studies followed by magnetic resonance imaging (MRI) in 10 studies and ultrasonography (US) in one study. Notably, only one study employed a combined approach using both CT and MRI (Supplementary Materials Table S1). The workflow of machine learning combined with radiomics facilitating the personal treatment of malignant liver tumors is depicted in Figure 1.

### 2.1. Development of Radiomics

Radiomics had its origins in 1999 when Gillies and his collaborators began exploring tumor heterogeneity, aiming to capture the variability in appearance, structure, and behavior [53]. In 2010, Gillies first introduced the concept of “radiomics” [54], and subsequently, Lambin and colleagues refined and defined it as the “high-throughput extraction of extensive image features from radiographic images” [55]. Radiomics for traditional imaging techniques like CT, MRI, and US unveiled layers of information beyond human perception, leading to the development of algorithms capable of extracting hundreds or thousands of image features. These attributes encompassed factors such as intensity, texture, wavelet, and fractal properties [2,3]. When coupled with machine learning, the analysis of these features became pivotal in correlating with clinical outcomes and tumor biology.

The most recent phase of radiomics development has witnessed the integration of radiomics with advanced computational methodologies, notably machine learning and deep learning. Machine learning empowers computers to glean insights from data and make predictive inferences, while deep learning deploys multi-layered artificial neural networks to decipher intricate patterns within extensive datasets. Deep learning networks encode medical images into shape information and abstract textural information through shallow and deep layers, as exemplified by Wang et al., who proposed a novel method for automatically extracting deep learning features from MR imaging, demonstrating their superior performance in predicting the malignancy of HCC [56].

Radiomics has flourished into a vibrant domain of medical research and practice, with its evolution continually driven by technological advancements and methodological refinements. Its transformative potential in enhancing our understanding and management of diseases through the prism of medical imaging continues to evolve and redefine the boundaries of medical science.

### 2.2. Procedures of Radiomics

Radiomics procedures adhere to a meticulously structured framework for the extraction and analysis of radiomic features from medical images. This framework encompasses the following key steps: image acquisition, image segmentation, feature extraction, feature selection, and feature analysis.

Firstly, the process commences with the acquisition of high-quality medical images through modalities like CT, MRI, or US. Emphasis is placed on optimizing image quality, ensuring high resolution, contrast, and signal-to-noise ratio to effectively capture relevant information. The application of resampling and intensity normalization is imperative to mitigate the impact of inconsistent imaging acquisition protocols and reconstruction procedures [57,58,59]. The subsequent step involves delineating the region of interest (ROI) from the background. This can be achieved through manual, semi-automatic, or automatic segmentation techniques [60]. Precision and reproducibility in ROI segmentation are essential, as they directly influence the accuracy of subsequent feature extraction and analysis. Thirdly, this pivotal stage entails extracting a diverse array of features from the segmented ROI. These features are derived using various methodologies, including shape analysis, first-order statistics, texture analysis, wavelet transforms, Laplacian transforms, and deep learning techniques [3]. The extracted features comprehensively encapsulate the heterogeneity and microenvironment complexity of the tumor. Fourthly, enhancing model performance and interpretability, it is necessary to select the most relevant and informative features from the initial pool of radiomics features. Methods such as correlation analysis, principal component analysis, and machine learning techniques are employed to reduce dimensionality and mitigate redundancy. This step focuses on the salient features for further analysis. Finally, the selected features undergo thorough analysis using statistical tests, clustering, classification, regression, or survival analysis. The objective is to construct radiomics models that unveil intricate relationships between these features and clinical outcomes or molecular characteristics of the tumor. These models play a crucial role in translating radiomic data into clinically relevant insights.

This rigorous procedural framework ensures the effectiveness and utility of radiomics in the realm of medical imaging and oncology. It establishes a foundation for the systematic extraction, analysis, and interpretation of radiomic information, contributing to enhanced decision making in clinical practice and research.

## 3. Radiomics Facilitating the Personal Treatment of Malignant Liver Tumors

In recent years, the fusion of radiomics and machine learning has yielded promising results in the evaluation of treatments for malignant liver tumors (e.g., surgical resection, liver transplant, ablation therapy, transarterial chemoembolization (TACE), radiotherapy, and systemic therapy [9,10,11,12,13,14,15,16,17,18,19,20,21,22,23,24,25,26,27,28,29,30,31,32,33,34,35,36,37,38,39,40,41,42,43,44,45,46,47,48,49,50,51,52]). Supplementary Materials Table S1 provides an overview of these significant contributions.

### 3.1. Surgical Therapy for Malignant Liver Tumors

Surgical therapy, encompassing liver resection and liver transplantation, represents the optimal approach for treating malignant liver tumors. Notably, postoperative recurrence is a critical prognostic factor, drawing the attention of clinicians.

For HCC, the prediction of recurrence has garnered considerable interest. Ji et al. [34] harnessed contrast-enhanced CT images from 470 patients with solitary HCC who underwent surgical resection to construct a recurrence prediction model using a machine-learning framework, achieving a concordance index (C-index) of 0.633–0.699. By integrating clinical features into the predictive model, superior prognostic performance was realized with a C-index of 0.733–0.801. Several similar studies employed various machine learning modeling methods, including random forest and SVM [20,25,27,50], to establish predictive models and achieved remarkable results in predicting HCC recurrence, with area under the receiver operator characteristic curves (AUCs) ranging from 0.834 to 0.948. These advancements facilitate a more precise evaluation of recurrence in HCC patients following surgical resection.

Deng et al. [43] demonstrated that radiomics models hold an advantage in predicting overall survival (OS) following radical resection. By constructing a radiomics prediction model using pre-surgery CT images, they achieved AUCs of 0.905, 0.884, and 0.911 for predicting 1-year, 3-year, and 5-year OS, respectively, in the validation cohort. This research may hold potential implications for informing clinical treatment decisions and prognostic assessments post surgery. Furthermore, the assessment of functional liver reserve before surgical resection is of critical importance, as it relates to the risk of post-hepatectomy liver failure [61]. Zhu et al. [32] utilized preoperative MRI and CT images to develop radiomics models for evaluating the functional liver reserve of HCC patients. In groups with indocyanine green retention rates at 15 min set at 10%, 20%, and 30%, the MRI radiomics model outperformed CT, with AUCs of 0.917 vs. 0.822, 0.979 vs. 0.860, and 0.961 vs. 0.938, respectively. Similarly, Wang and collaborators [51] employed preoperative gadoxetic acid-enhanced MRI radiomics features and an unsupervised machine learning approach to assess the risk of liver failure in HCC patients with varying functional liver reserves, revealing significant distinctions among functional liver reserve subgroups.

For ICC, Jolissaint et al. [28] retrospectively analyzed 138 ICC patients who underwent surgical resection, incorporating CT texture features and tumor size to predict early intrahepatic recurrence. The model achieved an AUC of 0.840 for recurrence prediction in the validation cohort. This result was successfully replicated in a multicenter study by Bo et al. [29], who enrolled 127 ICC patients undergoing curative surgery from three institutions. Their machine learning radiomics models, based on CT images, exhibited a mean AUC of 0.87 ± 0.02 for predicting early recurrence. Additionally, Qin et al. [35] conducted a retrospective study involving 274 perihilar cholangiocarcinoma patients who underwent contrast-enhanced CT and curative resection. They developed a multilevel predictive model that performed impressively in quantifying the risk of early recurrence, with AUCs reaching 0.883. The accuracy of the multilevel predictive model was 0.826, significantly surpassing the accuracy of conventional staging systems (0.641 for the American Joint Committee on Cancer 8th TNM, 0.617 for Memorial Sloan-Kettering Cancer Center, and 0.581 for Gazzaniga systems) [62,63,64].

Liver metastases are widespread, with colorectal metastases being the most common [65]. Granata et al. [16] harnessed radiomics features extracted from multiphase MR images, employing both traditional machine learning and deep learning frameworks to predict clinical outcomes in colorectal liver metastases (CLM) patients following liver resection. These features and models demonstrated significant prognostic value in evaluating recurrence, mutational status, pathological characteristics, and surgical resection margin, with accuracy ranging from 82% to 95%.

### 3.2. Nonsurgical Resection Therapy for Malignant Liver Tumors

Nonsurgical resection therapy serves as a vital complementary treatment for malignant liver tumors, encompassing therapies like ablation therapy, TACE, radiotherapy, and systemic therapy. The following are some radiomics studies associated with these treatment modalities.

#### 3.2.1. Ablation Therapy

Ablation therapy represents one of the radical treatment approaches for liver malignant tumors, particularly suitable for small HCC and liver metastases.

In the context of HCC, Tabari et al. [31] collected pre-ablation MR images to predict post-ablation pathologic treatment responses in early-stage HCC patients undergoing liver transplant. By constructing a radiomics model using machine learning, they discovered that pre-ablation MRI radiomics features could predict the pathologic treatment response of tumors in HCC patients undergoing ablation therapy, achieving an AUC of 0.830. Peng et al. [49] enrolled 149 HCC patients who underwent curative ablation with the goal of predicting recurrence-free survival. The random survival forest model, which integrated MRI radiomics and clinicopathological features, demonstrated strong prognostic value for evaluating early recurrence, with a C-index ranging from 0.733 to 0.801. This effort may hold potential in stratifying patients for the adoption of the most appropriate follow-up and intervention strategy.

In the case of liver metastases, Taghavi et al. [18] found that a CT radiomics model could predict local tumor progression for CLM before thermal ablation, with a C-index of 0.780. Their analysis included 90 CLM patients with 140 lesions. Shahveranova et al. [39] constructed a combined model based on MRI radiomics and clinical characteristics and arrived at a similar conclusion, with AUCs ranging from 0.927 to 0.981. Subsequently, Taghavi et al. [17] sought to validate whether radiomics features derived from pre-ablation CT images of patients with colorectal cancer could predict the development of new CLM after successful thermal ablation. However, they were unable to identify an effective predictive model, with AUCs ranging from 0.520 to 0.570, only achieving inferior performance in an external validation cohort.

#### 3.2.2. TACE

TACE is a common treatment for HCC and is particularly suitable for intermediate-stage HCC [61]. However, predicting the responses of HCC to TACE remains a challenge.

Liu et al. [9] used contrast-enhanced US cine images to predict the personalized response of HCC to the initial TACE treatment. They constructed a radiomics contrast-enhanced US model using deep learning, achieving an AUC of 0.930 in the validation cohort. In parallel, Shi et al. [15] and Peng et al. [38] each conducted single-center and multicenter studies to explore the ability of CT to predict the response of HCC to TACE. Remarkably, the radiomics models developed by these research teams achieved AUCs ranging from 0.949 to 0.994 in validation cohorts. Additionally, Bernatz et al. [21] found that a CT radiomics model could identify HCC patients responding to repetitive TACE, thereby contributing to the refinement of treatment algorithms. Similar studies [36,45] in the field of MRI combined with machine learning also reported promising results. These endeavors may contribute to selecting appropriate HCC patients who respond to TACE and enhancing the patient prognosis.

In terms of selecting suitable HCC patients for TACE, Wang et al. [11] retrospectively enrolled multicenter HCC patients who underwent TACE treatment. They found that a CT radiomics model could effectively discriminate between suitable and unsuitable HCC patients for TACE, achieving an AUC of 0.894 in the validation cohort.

For survival prognosis, Liu et al. [40] and Wang et al. [23] utilized CT images to develop CT radiomics-based survival prognosis models using a deep learning framework to predict the overall survival of HCC patients after TACE treatment. These models achieved C-index values ranging from 0.649 to 0.730. Although TACE in combination with tyrosine kinase inhibitor has been shown to improve outcomes in HCC patients, identifying patients who might benefit from the combined treatment remains challenging [66]. Ren et al. [44] recruited HCC patients who received the combined treatment and exacted radiomics and deep learning features from pretreatment CT images to constructed models for predicting outcomes, achieving AUCs ranging from 0.870 to 0.940. The results may offer a rapid and supportive method to identify patients likely to benefit from the combined treatment and have the potential to improve precision oncology.

In an effort to predict which HCC patients might develop extrahepatic spread or vascular invasion after initial TACE monotherapy, Jin et al. [33] retrospectively enrolled 256 patients and developed a combined model that integrated clinicoradiological predictors and a CT radiomics signature. The radiologic characteristics of tumors were evaluated by two experienced radiologists, blinded to patient information, who jointly reviewed all CT images to validate nine radiographic phenotypes, including (a) tumor number, (b) tumor size, (c) enhancement pattern. This combined model exhibited superior discrimination performance compared to the clinicoradiological model (AUCs 0.911 vs. 0.772 in the training set; AUCs 0.847 vs. 0.746 in the testing set). Importantly, it demonstrated the capacity to effectively stratify HCC patients based on their risk levels, potentially refining follow-up strategies for these patients.

These outstanding efforts underscore the enormous potential of radiomics in improving patient selection and treatment outcomes in the context of TACE.

#### 3.2.3. Radiotherapy

Radiotherapy is categorized into external radiotherapy, such as stereotactic body radiation therapy (SBRT), and internal radiotherapy, such as transarterial radioembolization (TARE). It is a common and suitable treatment for unresectable HCC and liver metastases.

Fontaine et al. [12] conducted a retrospective multicenter study utilizing both unsupervised and supervised clustering methods to construct an MRI radiomics model for predicting overall survival in HCC patients after SBRT. However, the model’s performance was suboptimal, with a sensitivity of 0.52 and specificity of 0.71. Some researchers have also dedicated their efforts to the study of liver metastases. Hu et al. [46] retrospectively acquired data from 97 CLM patients after SBRT and developed an automated model to predict progression-free survival using CT radiomics and machine learning, achieving a C-index of 0.68.

Stüber et al. [14] collected CT images from 491 CLM patients who underwent TARE to extract radiomics features and create models. Nevertheless, they did not observe significant additional prognostic value in these radiomics features for predicting overall survival when compared to information obtained solely from clinical parameters. Kobe et al. [42] aimed to predict treatment response to TARE in patients with liver metastases using pre-treatment CT images, employing both traditional machine learning and deep learning algorithms. The model achieved an AUC of 0.850, a sensitivity of 94.2%, and a specificity of 67.7% in a testing set.

Based on the findings from these studies, it is evident that the applications of radiomics combined with machine learning face several challenges in the field of liver malignant tumors after radiotherapy, particularly regarding the inferior performance of these models. Therefore, there is a pressing need for more advanced methods and innovative research in this area.

#### 3.2.4. Systematic Therapy

Systemic therapy encompasses various anti-tumor treatments, primarily including molecular targeted drug therapy, immunotherapy, and chemotherapy. Numerous researchers have been exploring the applications of radiomics combined with machine learning in the systemic treatment of liver malignant tumors, as follows.

In terms of HCC, several notable studies have leveraged radiomics and deep learning techniques. Tian et al. [10] developed a preoperative MRI model that integrated radiomics and deep learning features to predict the programmed death-ligand 1 (PD-L1) expression level in HCC patients. This model exhibited robust predictive performance, achieving an AUC of 0.897, surpassing the performance of the radiomics-only MRI model with an AUC of 0.794. Dong et al. [37] aimed to predict the efficacy of anti-programmed death-1 (PD-1) antibodies in combination with tyrosine kinase inhibitors for advanced HCC. Their CT radiomics model achieved an AUC of 0.792 in the testing cohort, and radiomic features were found to be associated with overall survival. Bo et al. [41] also constructed CT radiomics models to predict the response to lenvatinib monotherapy for unresectable HCC patients. In this retrospective multicenter study involving 109 patients, the optimal radiomics model achieved impressive AUCs of 0.970 in the training cohort and 0.930 in the external validation cohort. Similarly, in the case of ICC, Zhang et al. [30] used combined models based on MRI radiomics and clinical features to predict PD-1 and PD-L1 expression of ICC, achieving AUCs of 0.897 and 0.890, respectively.

With respect to CLM, which are typically treated with first-line chemotherapy, a subset of patients benefits from this standard treatment [67,68]. Giannini et al. [22] developed a CT delta-radiomics score to predict the response of CLM patients to first-line chemotherapy, achieving a sensitivity of 85% and a specificity of 92%. Qi et al. [19] employed artificial neural networks and machine learning algorithms to create a predictive model based on CT images and clinical features, identifying CLM responses to first-line chemotherapy with AUCs of 0.754 in the training cohort and 0.752 in the validation cohort. Additional studies [13,26] used radiomics to predict CLM responses to first-line chemotherapy, yielding favorable results.

Antiangiogenic drugs are increasingly combined with chemotherapy in CLM patients [69]. Qu et al. [24] used a dynamic radiomics feature extraction method to construct a CT radiomics model for predicting the efficacy of antiangiogenic therapy in CLM patients, achieving a promising AUC of 0.945 and accuracy of 0.855. To identify CLM patients sensitive to therapy targeting the anti-epidermal growth factor pathway, Dercle et al. [48] built a CT radiomics model using a deep learning and machine learning framework, achieving an AUC of 0.800 for predicting sensitivity.

In the context of HER2-amplified CLM patients, Giannini et al. [47] developed and validated a CT score to predict the response of individuals undergoing dual HER2-targeted therapy. The model effectively differentiated between responders and non-responders, with a sensitivity of 90% and a specificity of 42% in a validation dataset. This finding may have the potential to pave the way for a more aggressive diagnostic and dual HER2-targeted therapeutic approach in selected patients.

For patients with liver metastases from breast cancer, He et al. [52] investigated whether CT radiomics could predict the efficacy of anti-HER2 therapy, achieving an AUC of 0.865 for predicting the poor prognosis group. These studies collectively demonstrate that radiomics combined with machine learning serves as a powerful tool for personalizing the treatment of patients with liver malignant tumors in the context of systemic therapy.

## 4. The Future Opportunities and Challenges of Radiomics

While radiomics has demonstrated its significance in assessing the treatment of liver malignant tumors, it faces several challenges in its clinical application.

Firstly, many current radiomics studies have primarily emphasized model performance while overlooking the importance of study design quality and result analysis. Reliability in model validation is often compromised due to the use of random split validation methods and the absence of sample size calculations. These systemic errors may lead to falsely elevated model performance. This limitation hinders the translation of radiomics findings into practical clinical applications.

Secondly, there is a lack of biologically explainable analysis of radiomics features. Although some studies have employed algorithms like Shapley values to analyze the mathematical interpretability of input and output radiomics features [11], the biological interpretability of radiomics features still lags behind traditional radiological, histopathological, and molecular gene expression signatures [70]. Researchers often struggle to obtain clear logical explanations for results within the enigmatic “radiomics” black box.

Thirdly, the generalizability of radiomics models is challenging to ensure due to the absence of multicenter or prospective external validation. Radiomics, as a high-throughput feature extraction method, can generate thousands of features, resulting in a high-dimensional dataset that may produce spurious correlations with outcome events. A simulation study has demonstrated that significant features with prognostic value could even be found among randomly generated features [71]. As a result, rigorous multicenter spatial and prospective temporal external validations are urgently needed before radiomics can be applied in real-world clinical settings. Unfortunately, many studies currently lack this essential level of reliability and robustness in their validation processes.

In light of these challenges, future research must prioritize rigorous quality control in study design and result analysis, foster data sharing across multiple centers, and focus on intensive external validation involving diverse geographical regions and populations. Developing artificial intelligence algorithms with enhanced accuracy and interpretability is crucial to facilitate broader translation and clinical applications. Furthermore, considering the recent advancements in machine learning algorithms, deep learning has shown similar or even superior performance in evaluating therapeutic efficacy [9,10,23,36,38,40,44,48] and a wide range of clinical applications [72,73,74]. Therefore, exploring how to appropriately combine radiomics with deep learning for improved clinical individualization is a topic worthy of consideration.

## 5. Conclusions

In the assessment of liver malignant tumor treatments, the combination of radiomics and machine learning demonstrates substantial potential for improving therapeutic evaluation, enhancing predictive accuracy, supporting clinical decision making, and enabling personalized patient care. Nevertheless, the limitations of current research pose significant challenges to the widespread clinical adoption of radiomics. To advance the clinical application of radiomics, future research efforts should prioritize the quality control of study design and statistical analysis, biological interpretability of radiomics features and models, value of mature imaging models in real-world clinical treatment, and multicenter as well prospective clinical validations. In summary, radiomics combined with machine learning holds significant promise for reshaping the assessment and personalization of liver malignant tumor treatments. Addressing these research areas is instrumental in realizing the potential of radiomics for the benefit of both healthcare providers and patients.

## Data Availability

No new data were created or analyzed in this study. Data sharing is not applicable to this article.

## Supplementary Materials

The following supporting information can be downloaded at: https://www.mdpi.com/article/10.3390/biomedicines12010058/s1, Table S1: Summary of published radiomics studies on the realm of treating malignant liver tumors.
