# Supplementary material for: Machine Learning Combined with Radiomics Facilitating the Personal Treatment of Malignant Liver Tumors
Source: Biomedicines. 2023 Dec 26;12(1):58. doi: 10.3390/biomedicines12010058 (PMC10813632; doi:10.3390/biomedicines12010058)
Supplement: Supplementary file 1 [file biomedicines-12-00058-s001.zip › biomedicines-2755267-supplementary.pdf]

Supplementary Material

Table S1. Summary of published radiomics studies on the realm of treating malignant liver tumors.

Number	Reference	Study design (retrospective/prospective, single or multicenter study)	No. of patients	Type of tumors	No. and type of radiomic features	Statistical analysis (feature selection and modelling)	Imaging modality	Clinical Characteristics
1	Liu et al [9]	retrospective study, single center	130	HCC	Deep learning feature	Stochastic gradient descent solver, Cross-entropy loss function, Depthwise convolution, Global pooling, Video frame sampling, and Data augmentation	Ultrasound	Predicting responses to TACE
2	Tian et al [10]	retrospective study, single center	103	HCC	Deep learning + 1595 (intensity, geometry, gradient, texture, wavelet, square, square root, logarithm, exponential, Laplacian of Gaussian, and local binary pattern 3D)	Pearson correlation coefficie, recursive feature selection, SVM-Recursive Feature Elimination, and linear kernel function	MRI	Assessing PD-L1 Expression Level
3	Wang et al [11]	retrospective study, multicenter	243	HCC	First-order, shape, texture, wavelet and Laplacian of Gaussian	Spearman correlation analysis, LASSO, SVM, DT, RF, XGBoost, KNN, LDA, Adaboost, univariate and multivariate logistic regression analyses	CT	Identifying the suitability of TACE treatment in intermediate-stage HCC

4	Fontaine et al [12]	retrospective study, multicenter	81	HCC	39 (shape, First-order, Gradient-based, Second-order)	Unsupervised clustering (k-means, hierarchical clustering and Affinity Propagation), Supervised clustering(mRMR)	MRI	Predicting overall survival after radiotherapy for HCC
5	Defeudis et al [13]	retrospective study, single center	92	CLM	75 (intensity, histogram, and texture)	Genetic algorithm, Gaussian Naive Bayes classifier, Multilayer Perceptron, and SVM	CT	Predict response to first line chemotherapy in CLM
6	Stüber et al [14]	retrospective study, single center	491	CLM	1218 radiomics features	principal component analysis, a regression model with elastic net regularization, RSF, and XGBoost	CT	Predicting overall survival of CLM patients with radioembolization
7	Shi et al [15]	retrospective study, single center	164	HCC	851 radiomics features	LASSO, Logistic regression, SVM, multilayer perceptron, and RF	CT	Predicting the response to first TACE in intermediate-stage HCC
8	Granata et al [16]	retrospective study, multicenter	81	CLM	Deep learning + 851（first-order, shape, and texture）	univariate and multivariate, SVM, KNN, neural network and DT	MRI	Predicting clinical outcomes following liver resection in CLM patients

9	Taghavi et al [17]	retrospective study, single center	94	CLM	1767 (first-order, texture, Laplacian of Gaussian, wavelets, exponential, gradient, square, square-root, and logarithm)	Kruskal–Wallis test, Pairwise correlation, and RF	CT	Predict new liver metastasis after successful thermal ablation of CLM
10	Taghavi et al [18]	retrospective study, single center	90	CLM	105 (first-order, shape, and texture)	Inter-correlated features with pairwise correlation and Cox's proportional hazards model	CT	Predicting local tumor progression for CLM before thermal ablation
11	Qi et al [19]	retrospective study, single center	116	CLM	Deep learning + (shape, first-order, texture, Laplacian of Gaussian, wavelet, square, square root, logarithm and exponential)	Pearson correlation analysis, mRMR, Logistic regression, SVM, RF, AdaBoost, GBDT, and XGBoost.	CT	Identifying of CLM response to first-line irinotecan-based chemotherapy
12	Wu et al [20]	retrospective study, single center	132	HCC	396 (histogram, form factor, haralick features, and texture)	Analysis of variance, correlation analysis, LASSO, and gradient boosting DT	CT	Predicting early recurrence in HCC patients after partial hepatectomy
13	Bernatz et al [21]	retrospective study, single center	61	HCC	107 (shape, first-order, and texture)	LASSO, recursive feature elimination and recursive feature addition	CT	Identifying HCC patients who will respond to repetitive TACE

14	Giannini et al [22]	retrospective study, multicenter	242	CLM	107 (shape, intensity, and texture)	Mann–Whitney U test, SVM, Logistic regression, DT, and RF	CT	Predicting the response of individual CLM to first-line Oxaliplatin-based chemotherapy
15	Wang et al [23]	retrospective study, multicenter	543	HCC	Deep learning	LASSO, Cox regression analysis	CT	Evaluating survival prognosis of TACE in treatment-naïve patients with intermediate-stage HCC
16	Qu et al [24]	retrospective study, single center	76	CLM	first-order, shape, texture and wavelet	LASSO, SVM, LDA and RF	CT	Predicting the efficacy of antiangiogenic therapy in CLM
17	Wang et al [25]	retrospective study, single center	105	HCC	788 (shape, first-order, texture and wavelet)	LASSO	CT	Predicting the outcome of recurrence-free survival in HCC patients within three years after surgery
18	Giannini et al [26]	retrospective study, single center	95	CLM	22 (first-order and texture)	SVM	CT	Predicting response to chemotherapy of CLM

19	Shen et al [27]	retrospective study, single center	114	HCC	1044 (histogram, texture, form factor, and texture)	RF, Logistic regression	CT	Detecting early recurrence of HCC after resection or ablation
20	Jolissaint et al [28]	retrospective study, single center	138	ICC	254 (first-order, second-order, and texture)	mRMR, random forest.	CT	Predicting early liver recurrence after resection of ICC
21	Bo et al [29]	retrospective study, multicenter	127	ICC	107 (shape, first-order, and texture)	Independent-samples t test, mRMR, logistic, RF, neural network, Bayes, SVM, LightGBM, and XGBoost	CT	Predicting the early recurrence of ICC after curative resection
22	Zhang et al [30]	retrospective study, single center	98	ICC	396 (histogram, shape, and texture)	Spearman rank correlation, LASSO, and Logistic regression	MRI	Predicting PD-1/PD-L1 expression and outcome in ICC
23	Tabari et al [31]	retrospective study, single center	97	HCC	112 (shape, first-order, and texture)	mRMR, RF	MRI	Predicting pathologic treatment response in early-stage HCC patients who underwent ablation therapy

24	Zhu et al [32]	Retrospective study, single center	190	HCC	107 (first-order, shape, and texture)	spearman correlation analysis, LASSO, SVM, Extra-Trees, RF, LightGBM and XGBoost	CT+MRI	Evaluating the functional liver reserve before hepatectomy for liver cancer
25	Jin et al [33]	retrospective study, multicenter	256	HCC	1218 (shape, first-order, texture, Laplacian of Gaussian, and wavelet)	Pearson correlation coefficients, Analysis of variance and Linear discriminant analysis	CT	Predicting patients with unresectable HCC to develop extrahepatic spread or vascular invasion after initial TACE monotherapy
26	Ji et al [34]	retrospective study, multicenter	470	HCC	846 (first-order, textual, and wavelet)	mRMR, RSF, LASSO, Cox regression algorithm, and Unsupervised hierarchical clustering	CT	Predicting recurrence of HCC after resection
27	Qin et al [35]	retrospective study, multicenter	274	perihilar cholangiocarcinoma	first-order, gradient, texture and wavelet	Intraclass-correlation coefficient, Holm t test, Mutual information analysis, Pearson correlation coefficients, Spearman and Kendall.	CT	Predicting early recurrence in perihilar cholangiocarcinoma after curative resection
28	Chen et al [36]	retrospective study, single center	172	HCC	110 radiomic features	Deep learning + KNN, SVM, LASSO, and mRMR	MRI	Predicting response to TACE in HCC

29	Dong et al [37]	retrospective study, single center	55	HCC	1229 (first-order, shape, and texture)	intraclass correlation coefficients, LASSO, SVM, naïve Bayes, recursive partitioning and regression trees, conditional inference trees, RF, KNN, neuralnet, boosting, bagging, and logistics	CT	Identifying progressive disease in advanced HCC receiving second-line systemic therapy
30	Peng et al [38]	retrospective study, multicenter	310	HCC	Deep Learning + (texture, shape, size, and wavelet)	Recursive feature elimination, Linear, Logistic, SVM, Gradient boosting machine, RF and back propagation calculation	CT	Prediction of the initial treatment response to TACE in patients with HCC
31	Shahveranova et al [39]	retrospective study, single center	42	CLM	121 (shape, first order, and texture)	Spearman correlation analysis, LASSO and Logistic regression	MRI	Predicting local tumor progression after microwave ablation in CLM patients
32	Liu et al [40]	retrospective study, single center	243	HCC	Deep learning feature	Deep auto-encoder, Time-varying deep learning-based algorithms, Cox-Proportional Hazard	CT	Predicting prognostic risk factors in HCC with TACE
33	Bo et al [41]	retrospective study, multicenter	109	HCC	224 radiomics features	K-means clustering	CT	Predicting response to lenvatinib monotherapy for unresectable HCC

34	Kobe et al [42]	retrospective study, single center	36	liver metastases	Deep learning + 104 (first-order, histogram, shape, and texture)	Intraclass-correlation coefficient	CT	Predicting treatment response to TARE of liver metastases
35	Deng et al [43]	retrospective study, single center	150	HCC	first-order, shape, texture	LASSO, Univariate and multivariate Cox regression analyses	CT	Predicting the overall survival of HCC patients after radical hepatectomy
36	Ren et al [44]	retrospective study, single center	103	HCC	Deep learning + (first-order, shape, and second-order)	Zero median, Absolute deviation, Univariate analysis, Recursive feature addition	CT	Predicting short-term disease control and overall survival in HCC patients who received the TACE and tyrosine kinase inhibitor
37	Liu et al [45]	retrospective study, multicenter	140	HCC	1210 (first-order, shape, texture, Laplacian of Gaussian, and wavelet)	mRMR, LASSO	MRI	Predicting tumor response and outcome in HCC with TACE
38	Hu et al [46]	retrospective study, single center	97	CLM	108 (shape, intensity, and texture)	Variance inflation factor, Hazard ratios, and RSF	CT	Predicting progression-free survival for CLM treated with radiotherapy

39	Giannini et al [47]	retrospective study, single center	38	CLM	24 (shape, first-order, and texture)	Genetic algorithms	CT	Predicting behavior of individual CLM to HER2-targeted treatment
40	Dercle et al [48]	retrospective study, single center	667	CLM	Deep learning + 1757（first-order, shape, texture and wavelets）	Reproducibility analysis, Redundancy analysis, Feature ranking, SVM, KNN, Naive Bayes, RF, Bagging and LASSO	CT	Identifying CLM sensitive to therapies targeting EGFR pathway
41	Peng et al [49]	retrospective study, single center	149	HCC	1409 (first-order, shape, Laplacian of Gaussian, texture, and wavelet)	mRMR, RSF, SVM, Cox regression, RSF, and XGBoost	MRI	Predicting recurrence-free survival of HCC after curative ablation
42	Xie et al [50]	retrospective study, single center	259	HCC	101 (shape, first-order, and texture)	Spearman correlation test, Mann–Whitney U test, LASSO	CT	Assessing prognosis of early-stage HCC recurrence post-radical resection
43	Wang et al [51]	retrospective study, single center	276	HCC	107 (shape, first-order, and texture)	Intraclass-correlation coefficient, Spearman correlation analysis, Unsupervised clustering analysis	MRI	Identifying subgroups of patients with HCC with different liver function reserves

44	He et al [52]	retrospective study, multicenter	83	liver metastases from breast cancer	665 (first-order, shape, size, texture and wavelets)	Intraclass-correlation coefficient, Variance threshold, SelectKBest, LASSO, KNN, SVM, XGBoost, RF, LR, and DT	CT	Predicating the efficacy of anti-HER2 therapy for patients with liver metastases from breast cancer

Abbreviations: HCC: Hepatocellular carcinoma; ICC: intrahepatic cholangiocarcinoma; CLM: Colorectal liver metastases; TACE: Transarterial chemoembolization; CT: Computer tomography; MRI: Magnetic resonance imaging; TARE: Transarterial radioembolization; SBRT: Stereotactic body radiation therapy; DT: Decision tree; RF: random forest; XGBoost: Extreme gradient boosting; KNN: K-nearest neighbors; LDA: Linear discriminant analysis; SVM: Support vector machine; LASSO: Least absolute shrinkage and selection operator; mRMR: Minimum redundancy maximum correlation; RSF: Random survival forest; LightGBM: Light Gradient Boosting Machine; PD-1: Programmed death-1; PD-L1: Programmed death-ligand 1.
